# *Lactobacillus delbrueckii* subsp. *bulgaricus* Alleviates Acute Injury in Hypoxic Mice

**DOI:** 10.3390/nu16101465

**Published:** 2024-05-13

**Authors:** Ke Song, Hui Ling, Linlin Wang, Peijun Tian, Xing Jin, Jianxin Zhao, Wei Chen, Gang Wang, Yujing Bi

**Affiliations:** 1 State Key Laboratory of Food Science and Resources, Jiangnan University, Wuxi 214122, China; 6210112157@stu.jiangnan.edu.cn (K.S.); wanglinlin@jiangnan.edu.cn (L.W.); pjtian@jiangnan.edu.cn (P.T.); 8202008362@jiangnan.edu.cn (X.J.); zhaojianxin@jiangnan.edu.cn (J.Z.); chenwei66@jiangnan.edu.cn (W.C.); 2 School of Food Science and Technology, Jiangnan University, Wuxi 214122, China; 3 State Key Laboratory of Pathogen and Biosecurity, Beijing Institute of Microbiology and Epidemiology, Beijing 100071, China; 18375347133@163.com; 4 National Engineering Research Center for Functional Food, Jiangnan University, Wuxi 214122, China; 5 (Yangzhou) Institute of Food Biotechnology, Jiangnan University, Yangzhou 225004, China

**Keywords:** *Lactobacillus delbrueckii* subsp. *bulgaricus*, acute hypoxic injury, intestinal barrier, gut microbiota

## Abstract

Acute mountain sickness (AMS) is a common ailment in high-altitude areas caused by the body’s inadequate adaptation to low-pressure, low-oxygen environments, leading to organ edema, oxidative stress, and impaired intestinal barrier function. The gastrointestinal tract, being the first to be affected by ischemia and hypoxia, is highly susceptible to injury. This study investigates the role of *Lactobacillus delbrueckii* subsp. *bulgaricus* in alleviating acute hypoxic-induced intestinal and tissue damage from the perspective of daily consumed lactic acid bacteria. An acute hypoxia mouse model was established to evaluate tissue injury, oxidative stress, inflammatory responses, and intestinal barrier function in various groups of mice. The results indicate that strain 4L3 significantly mitigated brain and lung edema caused by hypoxia, improved colonic tissue damage, and effectively increased the content of tight junction proteins in the ileum, reducing ileal permeability and alleviating mechanical barrier damage in the intestines due to acute hypoxia. Additionally, 4L3 helped to rebalance the intestinal microbiota. In summary, this study found that *Lactobacillus delbrueckii* subsp. *bulgaricus* strain 4L3 could alleviate acute intestinal damage caused by hypoxia, thereby reducing hypoxic stress. This suggests that probiotic lactic acid bacteria that exert beneficial effects in the intestines may alleviate acute injury under hypoxic conditions in mice, offering new insights for the prevention and treatment of AMS.

## 1. Introduction

Acute mountain sickness consists of nonspecific symptoms that occur at altitudes above 2500 m. Individuals ascending to a new elevation may experience symptoms if they are not yet acclimated; these symptoms typically resolve automatically after appropriate measures are taken [1]. Many studies have shown that high altitudes can impair intestinal barrier function, primarily manifested by increased intestinal permeability [2], damaged intestinal villi [3], and inflammation [4]. Exposure to the low-pressure, low-oxygen environment at an altitude of 4000 m is sufficient to cause damage to rat intestinal epithelial mucosa and villi, leading to erythrocyte exudation [5]. Moreover, hypoxic environments can also damage the mucosal immune function of the intestines, resulting in an increase in pathogens in feces and aggravating pathological damage to colonic tissues [6]. Even for mountaineers who have some level of adaptation to high-altitude environments, high altitudes can induce gastrointestinal mucosal lesions [7]. In conclusion, the hypoxic environment of high altitudes can damage intestinal barrier function, disrupt the intestinal microenvironment, and further harm the body.

The gut microbiota exhibits a high degree of plasticity and is easily influenced by external environmental factors. Hypoxic conditions have a rapid and lasting impact on the human gut microbiota [8], and low-pressure hypoxia has been shown to have a strong and persistent effect on the gut microbial community in rats [9]. Altitude significantly affects the diversity and richness of gut microbiota in Tibetans, as well as altering the composition of the microbial community, primarily reflected in changes in the relative abundance of Firmicutes and Bacteroidetes [10]. A higher ratio of Firmicutes to Bacteroidetes may help the host absorb energy more efficiently [11]. Furthermore, the abundance of various genera, including *Lactobacillus* and *Streptococcus*, appears to be related only to altitude and duration of stay, with minimal influence from diet [2]. The abundance of *Prevotella* seems to increase with altitude, a trend observed in both pika populations and the gut microbiota of Han Chinese [12,13,14]. The gut microbiota serves as a biological barrier that maintains the intestinal environment’s balance and hinders pathogenic bacteria and toxins, playing a crucial role in individual health. Changes in gut microbiota may further compromise the intestinal barrier and trigger inflammation [15]. Overall, high-altitude environments profoundly affect the host’s gut microbiota, which appears to be related only to altitude, suggesting that the gut microbiota may be a potential intervention target for high-altitude diseases.

Probiotics have emerged as a dietary supplement in recent years. The Food and Drug Administration (FDA) and the World Health Organization (WHO) define probiotics as “live microorganisms that, when administered in adequate amounts, confer a health benefit on the host”. Dou et al. [16] found that *Lactobacillus casei* synthesizing selenium nanoparticles could effectively alleviate damage to intestinal barrier function under acute low-pressure hypoxic stress; *Lactobacillus johnsonii* was able to reduce potential endogenous pathogens at high altitudes, thereby preventing gastrointestinal dysfunction, with further research suggesting this preventative effect may be related to the regulation of intestinal damage [17]. Synbiotics, a combination of probiotics and prebiotics, have been proven to alleviate rat cardiac hypertrophy caused by low-pressure hypoxia [18] and improve intestinal inflammation [19]. The protective effects of probiotics on the gut are undeniable. However, there is still limited research on whether commonly consumed lactic acid bacteria have the potential to alleviate acute hypoxic injury. The *Lactobacillus delbrueckii* subsp. *bulgaricus* is a common lactic acid bacterium used in yogurt. Yogurt is a traditional food commonly consumed in high-altitude regions of China. Therefore, this study on the role of *Lactobacillus delbrueckii* subsp. *bulgaricus* in alleviating altitude-related injuries will help to prove whether yogurt could be used as a potential dietary intervention to alleviate altitude-related diseases.

## 2. Materials and Methods

### 2.1. Materials and Reagents

*Lactobacillus delbrueckii* subsp. *bulgaricus* was obtained from the Culture Collection Bank of the School of Food Science and Technology at Jiangnan University. Detailed information about the strain is listed in Table 1. Skim milk powder was purchased from New Zealand Milk Brands Limited (Auckland, New Zealand); TRIZOL reagent was obtained from Thermo Fisher Scientific (Waltham, MA, USA); DEPC-treated water and RT-qPCR primers were acquired from Sangon Biotech Co., Ltd. (Shanghai, China); reverse transcription kits, RNA extraction kits, and qPCR fluorescent dyes were sourced from Novozan Biotech Co., Ltd. (Nanjing, China); fecal DNA extraction kits were purchased from MP Biomedicals (Santa Ana, CA, USA); DNA Gel Extraction Kits were bought from Biomiga (San Diego, CA, USA); ELISA kits for tumor necrosis factor-α (TNF-α), interleukin-6 (IL-6), interferon-γ (IFN-γ), zonula occludens 1 (ZO-1), occludin, claudin-1, hypoxia inducible factor-1α (HIF-1α), erythropoietin (EPO), vascular endothelial growth factor (VEGF), malondialdehyde (MDA), superoxide dismutase (SOD), diamine oxidase (DAO), and glutathione peroxidase (GSH-Px) were obtained from Senbeijia Biotechnology Co., Ltd. (Nanjing, China); BCA protein assay kits were sourced from Beyotime Biotechnology (Shanghai, China).

The strain of *Lactobacillus delbrueckii* subsp. *bulgaricus* was streaked on solid MRS medium and incubated at 37 °C for 48 h. Single colonies were selected and inoculated into liquid MRS medium, followed by incubation at 37 °C for 18 h without agitation. The strains were then continuously cultured three times with a 1% (*v*/*v*) inoculum. The collected cells (8000× *g*, 5 min, 4 °C) were washed twice with sterile normal saline and then resuspended to 1 × 10^10^ colony-forming units/mL for gavage.

### 2.2. Animal Experiment Design

The animal experiment protocol was approved by the Medical Ethics Committee of the Military Medical Research Institute with approval number: IACUC-DWZX-2023-020. The experiments were strictly conducted in accordance with the National Research Council’s Guide for the Care and Use of Laboratory Animals. Forty-eight 8-week-old male specific pathogen-free C57BL6/J mice (20 ± 1 g) were purchased from Vital River Laboratory Animal Technology Co., Ltd. (Beijing, China). The mice were randomly divided into eight groups with six mice per cage. The groups were labeled as follows: control group (Control, *n* = 6), hypoxia group (HH, *n* = 6), acetazolamide group (AC, *n* = 6), *Lactobacillus delbrueckii* subsp. *bulgaricus* 4L3 group (4L3, *n* = 6), *Lactobacillus delbrueckii* subsp. *bulgaricus* 3L9 group (3L9, *n* = 6), *Lactobacillus delbrueckii* subsp. *bulgaricus* 2L1 group (2L1, *n* = 6), *Lactobacillus delbrueckii* subsp. *bulgaricus* 1L2 group (1L2, *n* = 6), and *Lactobacillus delbrueckii* subsp. *bulgaricus* 5L6 group (5L6, *n* = 6). The number of animals in each group was determined based on the conditions of the experimental equipment and to meet statistical requirements.

**Figure 1:**
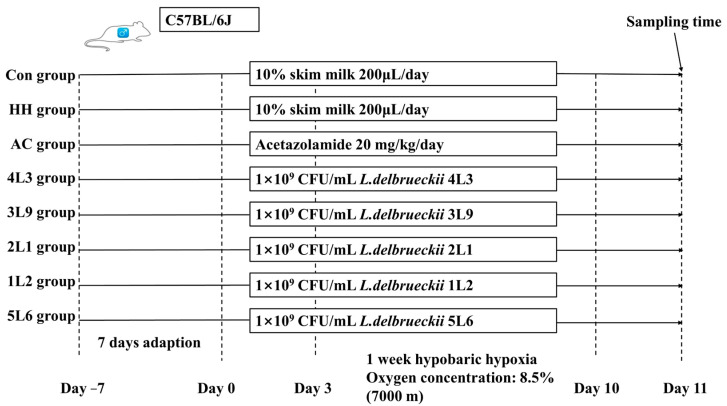
Animal experimental protocol.

In the first week of the experiment, the eight groups of mice were allowed to feed and drink freely for acclimatization. Once the experiment commenced, the control and model groups were gavaged daily with 200 μL of 10% skim milk, while the AC group received 20 mg/kg body weight of acetazolamide via gavage. The *Lactobacillus* intervention groups were each gavaged with 200 μL of a 1.0 × 10^10^ CFU/mL bacterial suspension. Three days after gavage initiation, all groups except the control were transferred to a hypoxic animal chamber for a 7-day exposure to low oxygen conditions at an oxygen concentration of 8.5%, the atmospheric pressure was at standard level, during which time gavage with acetazolamide and *Lactobacillus* suspension was maintained. Throughout the animal trial, daily recordings of mouse body weight were taken. At the end of the experiment, mice were anesthetized with isoflurane, blood samples were collected via eyeball extraction, and the mice were euthanized by cervical dislocation. Relevant tissue samples were then rapidly transferred to liquid nitrogen for preservation for subsequent experimental index measurements. The specific experimental protocol is shown in Figure 1.

Acetazolamide is currently the only medicine approved by the US FDA for the prevention and treatment of acute mountain sickness. The selection of its dosage is based on existing research reports [20]. The intervention dosage of *Lactobacillus* chosen in this study (200 μL of a 1.0 × 10^10^ CFU/mL) was based on previous experimental reports (1.0 × 10^9^ CFU) [19], and adjustments were made based on our laboratory experience to ensure that the viable count of live bacteria administered to each mouse per gavage exceeded 1.0 × 10^9^ CFU. The determination of intervention time and altitude was based on previous similar studies [16,19].

### 2.3. Body Weight and Organ Water Content

Mouse body weight was recorded daily during the experiment. After the experiment, mouse brain and lung tissues were wiped with absorbent paper to remove surface blood and water, then immediately weighed (wet weight). The brain and lung tissues were dried in an oven at 70 °C to a constant weight (dry weight), and water content was calculated as (wet weight−dry weight)/wet weight.

### 2.4. Histological Analysis of Colon, Lung, and Brain Tissues

The colon, brain hippocampus region, and lung tissues were fixed in tissue fixative (4% paraformaldehyde). After 24 h of fixation, paraffin sections of mouse colon, hippocampus, and lung were prepared and stained with H&E. Pathological damage to the colon, brain, and lung tissues was observed using a Panoramic MIDI digital slide scanner (3DHISTECH Ltd., Budapest, Hungary).

### 2.5. Determination of Gene Transcription Levels in Colon Tissue

RT-qPCR was used to measure changes in mRNA levels of tight junction protein-related genes in mouse colon tissues. Total RNA from the colon was extracted using TRIzol, and RNA was reverse-transcribed into cDNA using a reverse transcription kit (R333-01). Real-time quantitative PCR reactions were performed using qPCR fluorescent dye (Q712-02) with the aforementioned cDNA as a template. The primers for related genes are listed in Table 2, with β-actin as the internal reference gene. The relative changes in target gene mRNA levels were calculated using the 2^−ΔΔCT^ method.

### 2.6. Integrity Measurement of Ileal Mechanical Barrier

A 1 cm section of ileum was taken, added to 500 μL of sterile PBS along with three sterile zirconia beads, and homogenized using a high-throughput tissue homogenizer (65 Hz, 30 s, 6 times). After centrifugation for 10 min (5000 rpm, 4 °C), the supernatant was collected and total protein content was determined according to the instructions of the Beyotime BCA Protein Assay Kit. The contents of DAO, occludin, ZO-1, and claudin-1 in ileum tissue supernatant as well as serum DAO levels were measured according to the instructions provided by the Nanjing Senbeijia Biological Technology Co., Ltd. (Nanjing, China).

### 2.7. Cytokine Measurement

Proteins were extracted from mouse ileum, and IL-6, IFN-γ, and TNF-α levels in the ileum tissue supernatant were measured according to the manufacturer’s instructions.

### 2.8. Antioxidant Capacity Assessment

Proteins were extracted from the mouse ileum, and the contents of MDA, GSH-Px, and SOD in the ileum tissue supernatant were measured according to the instructions provided by the kit.

### 2.9. Hypoxia-Related Factors Measurement

The levels of EPO, VEGF, and HIF-1α in mouse serum were determined following the instructions of the assay kits provided by Nanjing Senbeijia Biological Technology Co., Ltd.

### 2.10. Mouse Fecal Microbiota 16S rRNA Amplicon Sequencing

Total DNA was extracted from mouse feces using the MP rapid DNA extraction kit, and the V3–V4 variable regions of the bacterial 16S rRNA gene were amplified using forward primer 341F (5′-CTAYGGGRBGCASCAG-3′) and reverse primer 806R (5′-CTACNNGGGTATCTAAT-3′). The amplification products were subjected to electrophoresis on a 1.8% agarose gel, and the target DNA bands around 465 bp were excised and transferred to sterile centrifuge tubes for purification according to the gel recovery kit instructions. The purified DNA was sequenced on an Illumina MiSeq PE300 platform.

### 2.11. Mouse Fecal Metabolite Measurement

After freeze-drying the feces, 20 mg of the sample was weighed and homogenized in 200 μL of ultrapure water. Then, 800 μL of methanol–acetonitrile mixture (1:1) was added to precipitate proteins. The mixture was vortexed for 30 s, followed by ultrasonication in an ice-water bath for 10 min. After ultrasonication, the samples were incubated at −20 °C for 1 h to enhance protein precipitation. After centrifugation for 15 min (4 °C, 15,000 rpm), the supernatant was vacuum-concentrated until dry. Finally, 100 μL of acetonitrile-water (1:1) was added to reconstitute the sample, which was then vortexed for 30 s and centrifuged for 15 min (4 °C, 15,000 rpm). The supernatant was collected for fecal metabolite analysis.

### 2.12. Statistical Analysis

Data were statistically analyzed using GraphPad Prism version 10.0.2 (GraphPad Software, San Diego, CA, USA). The results are presented as mean ± standard error. Differences between groups were assessed using one-way analysis of variance (ANOVA), with Dunnett’s test and Wilcoxon rank-sum test and Linear discriminant analysis Effect Size (LEfSe) for analyzing microbiome differences. Spearman’s correlation coefficient was used to measure the correlation between factors. Differences between the model group and control group are indicated by #, while differences between intervention groups and model group are indicated by *. A *p*-value < 0.05 was considered statistically significant, with ^#^ *p <* 0.05; ^##^ *p <* 0.01; ^###^ *p <* 0.001; * *p <* 0.05; ** *p <* 0.01; *** *p <* 0.001.

## 3. Results

### 3.1. Lactobacillus delbrueckii subsp. bulgaricus 4L3 Significantly Alleviates Brain and Lung Edema in Mice

**Figure 2:**
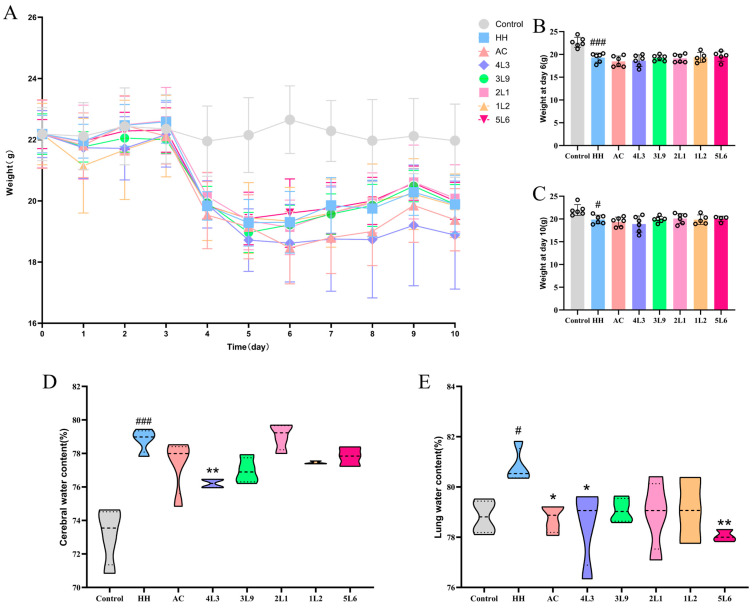
Effects of *Lactobacillus delbrueckii* subsp. *bulgaricus* on mouse body weight and organ water content. (**A**) Body weight (*n* = 6). (**B**) Body weight at day 6 (*n* = 5–6). (**C**) Body weight at day 10 (*n* = 5–6). (**D**) Brain water content (*n* = 3–4). (**E**) Lung water content (*n* = 3–4). Data are presented as mean ± SD and analyzed using ANOVA and Dunnett’s test. ^#^ indicates differences between the HH group and the control group, ^#^ *p <* 0.05; ^###^ *p <* 0.001. * indicates differences between the intervention groups and the HH group, * *p <* 0.05; ** *p <* 0.01.

In this study, a hypoxia-induced mouse model was established using a low-pressure oxygen chamber to simulate a 7000 m altitude hypoxic environment. The mice experienced a rapid weight loss (Figure 2A) with a decrease of about 2 g within one day of entering the low-oxygen chamber compared to the control group, and this weight loss continued for about three days, and reached its lowest point on day 6 (Figure 2B). Subsequently, the weight of the mice began to stabilize and recover, with no significant differences in weight between the intervention groups. On day 10, the HH group still lost a significant amount of weight compared to the control group (*p <* 0.05) (Figure 2C), but recovered compared to day 6. The water content in the brains of mice treated with hypoxia significantly increased (*p <* 0.001) (Figure 2D), while intervention with 4L3 significantly reduced the water content in the mouse brain (*p <* 0.05). The water content in the lungs of mice also significantly increased after hypoxia treatment (*p <* 0.05) (Figure 2E), and the AC group, 4L3 group, and 5L6 group were able to significantly reduce the water content in the mouse lungs (*p <* 0.05). The results suggest that 4L3 can significantly alleviate brain and lung edema caused by hypoxia treatment in mice. Although 5L6 has a significant effect in alleviating lung edema, it did not show a significant effect in alleviating brain edema.

### 3.2. Intervention with Certain Strains of Lactobacillus delbrueckii subsp. bulgaricus Alleviates Specific Tissue Damage in Mice

**Figure 3:**
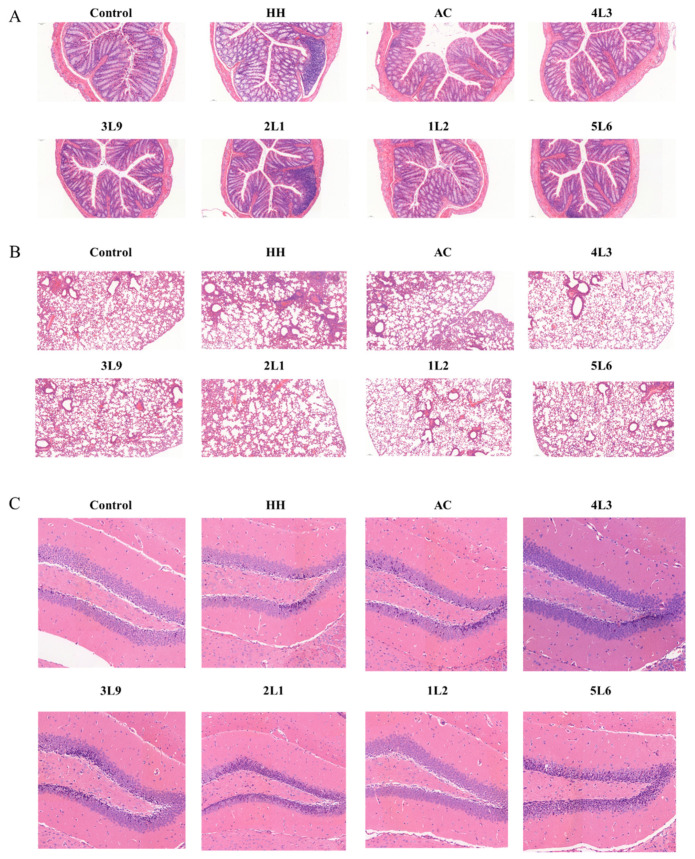
Impact of *Lactobacillus delbrueckii* subsp. *bulgaricus* on mouse tissue morphology. (**A**) Colon, magnification 400×. (**B**) Lung, magnification 200×. (**C**) Hippocampal DG region, magnification 400×.

After hypoxia treatment, the colonic crypts of mice were significantly atrophied with numerous vacuoles, reduced intestinal glands, and extensive infiltration of inflammatory cells (Figure 3A), indicating that the hypoxia treatment caused damage to the colonic tissue of mice. The intervention effects of different strains of *Lactobacillus delbrueckii* subsp. *bulgaricus* varied significantly. The infiltration of inflammatory cells was noticeably improved in the 4L3 and 1L2 groups, and the vacuoles in the crypts were significantly reduced compared to the model group. However, the 2L1 strain exacerbated colonic tissue damage to some extent, as evidenced by extensive infiltration of inflammatory cells. The HH group mice showed damage to alveolar structures with intra-alveolar hemorrhage, thickened alveolar walls, and slight infiltration of inflammatory cells (Figure 3B). The interventions with AC group, 4L3 group, and 1L2 group were more effective, with lung tissue morphology closely resembling that of the control group. In the hippocampal DG region of HH group mice, neuronal shrinkage and intensified cell staining were observed (Figure 3C). AC and 4L3 treatments could not reduce the impact of hypoxia on mouse brains, only the 1L2 strain was able to reverse the effects of hypoxia on the hippocampal DG region of mice. Overall, strains 4L3 and 1L2 were able to effectively alleviate damage to the colon and lungs of mice, with 1L2 also alleviating damage to the hippocampal DG region caused by hypoxia, indicating that there are differences in the intervention effects of different strains.

### 3.3. Influence of Lactobacillus delbrueckii subsp. bulgaricus on the Transcription Levels of Tight Junction Protein Genes in the Colon

**Figure 4:**
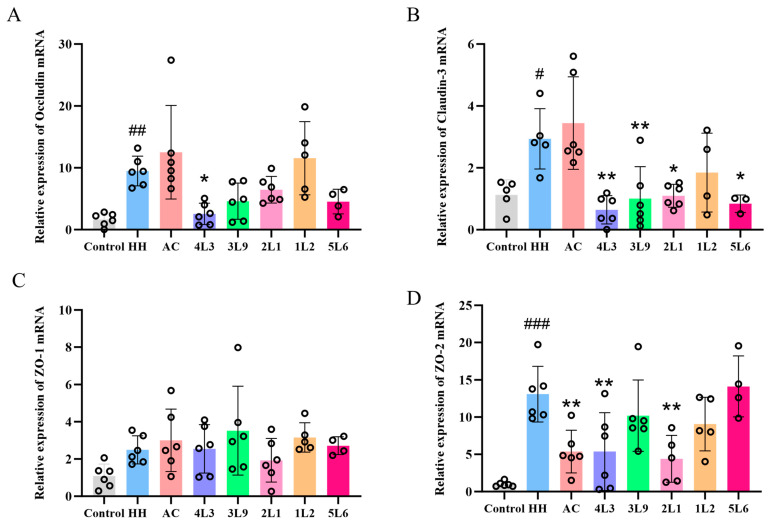
Effects of *Lactobacillus delbrueckii* subsp. *bulgaricus* on transcript levels of tight junction proteins in the mouse colon. (**A**) Colon occludin; (**B**) colon claudin-3; (**C**) colon ZO-1; (**D**) colon ZO-2 mRNA expression levels (*n* = 4–5). Data are presented as mean ± SD and analyzed using ANOVA and Dunnett’s test. ^#^ indicates differences between the HH group and the Control group, ^#^ *p <* 0.05; ^##^ *p <* 0.01; ^###^ *p <* 0.001. * indicates differences between the intervention group and the HH group, * *p <* 0.05; ** *p <* 0.01.

To investigate the effects of *Lactobacillus delbrueckii* subsp. *bulgaricus* intervention on the intestinal barrier function in mice, we examined the changes in mRNA expression levels of four tight junction proteins, occludin, claudin-3, ZO-1, and ZO-2, in mouse colon tissue. Compared with the control group, the expression levels of occludin, claudin-3, and ZO-2 mRNA were significantly increased in the HH group’s colon (*p <* 0.05) (Figure 4A,B,D). The expression level of occludin mRNA was significantly downregulated only after intervention with the 4L3 strain (*p <* 0.05), with no significant downregulation observed in other intervention groups. The 4L3, 3L9, 2L1, and 5L6 groups could significantly downregulate the expression level of claudin-3 mRNA in colon tissue (*p <* 0.05) (Figure 4B), while the AC, 4L3, and 2L1 groups could significantly downregulate ZO-2 mRNA expression (*p <* 0.05) (Figure 4D). Hypoxia exposure and lactobacilli intervention had no significant effect on the mRNA expression level of ZO-1 in colon tissue (Figure 4C). Overall, the expression levels of occludin, claudin-3, and ZO-2 mRNA in the colon tissue of the 4L3 group were significantly downregulated, approaching those of the control group, suggesting that the colon barrier damage was less severe after 4L3 intervention.

### 3.4. Effect on Ileal Mechanical Barrier Integrity

**Figure 5:**
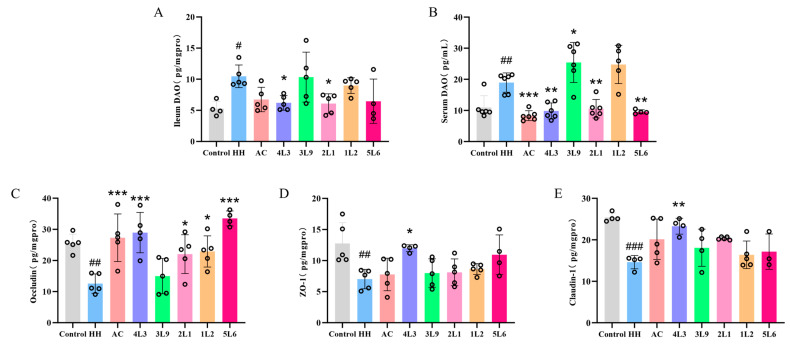
Effects of *Lactobacillus delbrueckii* subsp. *bulgaricus* on mouse intestinal barrier function of the mouse ileum. (**A**) Ileal DAO (*n* = 4–5). (**B**) Serum DAO (*n* = 4–6). (**C**) Ileal occludin, (**D**) Ileal ZO-1, (**E**) Ileal claudin-1 protein content (*n* = 4–5). Data are presented as mean ± SD and analyzed using ANOVA and Dunnett’s test. ^#^ indicates differences between the HH group and the control group, ^#^ *p <* 0.05; ^##^ *p <* 0.01; ^###^ *p <* 0.001. * indicates differences between the intervention group and the HH group, * *p <* 0.05; ** *p <* 0.01; *** *p <* 0.001.

To further explore the impact of the *Lactobacillus delbrueckii* subsp. *bulgaricus* intervention on the ileal barrier in mice, we measured the levels of DAO in the mouse ileum and serum (Figure 5A,B). The results showed that the levels of DAO in both ileum and serum were significantly increased in the HH group (*p <* 0.05), indicating that acute hypoxia treatment caused severe damage to the mechanical barrier of the mouse ileum. After intervention, the levels of DAO in the serum were significantly downregulated in the AC, 4L3, 2L1, and 5L6 groups (*p <* 0.01), and the levels of DAO in the ileum were also significantly reduced in the 4L3 and 2L1 groups (*p <* 0.05). The AC and 5L6 groups showed a downward trend in DAO levels in the ileum but these were not significant (*p* = 0.0777, *p* = 0.0719), indicating that strains 4L3 and 2L1 had a stronger protective effect on the ileal mechanical barrier. Further examination of the levels of tight junction proteins occludin, ZO-1, and claudin-1 in the ileum revealed that all three proteins were significantly decreased in the HH group’s ileum (*p <* 0.01) (Figure 5C–E), confirming that hypoxic conditions affect the tight junctions between ileal cells, thereby damaging the intestinal barrier. After intervention, all the levels of all three tight junction proteins were significantly upregulated in the ileum of the 4L3 group (*p <* 0.05), while Occludin levels were significantly upregulated in the AC, 2L1, 1L2, and 5L6 groups (*p <* 0.05), with no significant effect on ZO-1 and claudin-1 proteins. The 3L9 group did not significantly upregulate any of the three tight junction proteins. These results suggest that the *Lactobacillus delbrueckii* subsp. *bulgaricus* strain 4L3 can effectively protect against intestinal barrier damage caused by acute hypoxia in mice, while strain 3L9 has no protective effect, indicating significant differences in mitigation effects among different strains.

### 3.5. Lactobacillus delbrueckii subsp. bulgaricus Reduces Cytokine Secretion in the Ileum

**Figure 6:**
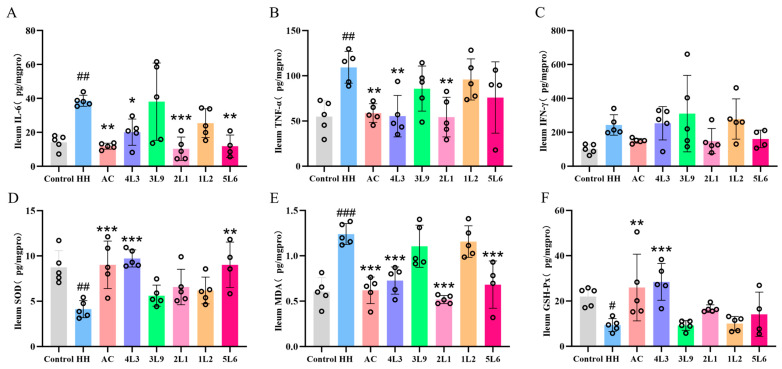
Effects of *Lactobacillus delbrueckii* subsp. *bulgaricus* on ileal cytokines and oxidative stress in mice. (**A**) Ileum IL-6; (**B**) ileum TNF-α; (**C**) ileum IFN-γ; (**D**) ileum SOD; (**E**) ileum MDA; (**F**) ileum GSH-Px (*n* = 4–6). Data are presented as mean ± SD and analyzed using ANOVA and Dunnett’s test. ^#^ indicates differences between the HH group and the control group, ^#^ *p <* 0.05; ^##^ *p <* 0.01; ^###^ *p <* 0.001. * indicates differences between the intervention group and the HH group, * *p <* 0.05; ** *p <* 0.01; *** *p <* 0.001.

Cytokine levels in mouse ileum tissue were measured, revealing that compared to the control group, the HH group had significantly increased levels of IL-6 and TNF-α (*p <* 0.01) (Figure 6A,B). After AC, 4L3, 2L1, and 5L6 interventions, the level of IL-6 in the ileum was significantly reduced (*p <* 0.05) (Figure 6A). After AC, 4L3, and 2L1 interventions, the level of TNF-α in the ileum was significantly reduced (*p <* 0.01) (Figure 6B). However, the level of IFN-γ in the mouse ileum did not significantly increase after acute hypoxic exposure, and the intervention groups had no significant effect on the level of IFN-γ in the ileum (Figure 6C). These results suggest that AC, 4L3, 2L1, and 5L6 can effectively mitigate the rise in IL-6 levels induced by hypoxia in mice and that AC, 4L3, and 2L1 can also effectively reduce the level of TNF-α in the mouse ileum, indicating potential for regulating inflammation responses induced by hypoxia.

### 3.6. Lactobacillus delbrueckii subsp. bulgaricus Alleviates Oxidative Stress in Mice

The levels of antioxidant factors in mouse ileum tissue were assessed, showing that compared to the control group, the HH group had a significant decrease in SOD and GSH-Px levels (*p* < 0.05) (Figure 6D,F), while MDA levels significantly increased (*p* < 0.001) (Figure 6E), indicating that acute hypoxic treatment severely affects the redox balance in mice, leading to an increase in reactive oxygen species. AC and 4L3 treatments could significantly reverse the changes in SOD, GSH-Px, and MDA levels induced by hypoxia (*p* < 0.01), while the 5L6 treatment significantly increased SOD levels (*p* < 0.01) and significantly decreased MDA levels (*p* < 0.001), but had no significant effect on GSH-Px levels. The MDA levels were significantly reduced in the 2L1 group (*p* < 0.001), but there was no significant effect on the SOD and GSH-Px levels. These results indicate that AC and 4L3 have a good mitigating effect on oxidative stress induced by acute hypoxia in mice.

### 3.7. Effect of Lactobacillus delbrueckii subsp. bulgaricus Intervention on Serum Hypoxia-Related Factors

**Figure 7:**
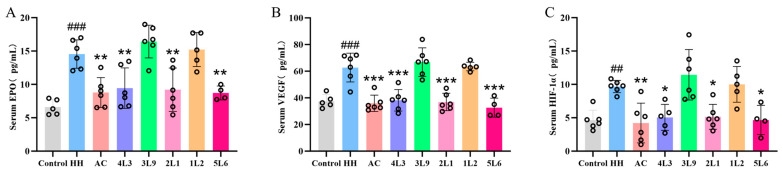
Effects of *Lactobacillus delbrueckii* subsp. *bulgaricus* on serum hypoxia-related factors in mice. (**A**) Serum EPO; (**B**) serum VEGF; (**C**) serum HIF-1α (*n* = 4–6). Data are presented as mean ± SD and analyzed using ANOVA and Dunnett’s test. ^#^ indicates differences between the HH group and the control group, ^##^ *p <* 0.01; ^###^ *p <* 0.001. * indicates differences between the intervention groups and the HH group, * *p <* 0.05; ** *p <* 0.01; *** *p <* 0.001.

Further examination of hypoxia-related factors in mouse serum revealed that hypoxic treatment significantly increased the levels of EPO, VEGF, and HIF-1α in mouse serum (*p <* 0.01) (Figure 7A–C), while after AC, 4L3, 2L1, and 5L6 treatments, the levels of all three factors were significantly reduced (*p <* 0.05), suggesting a reduction in hypoxic stress in mice. The consistency between serum hypoxia factor levels and ileal mechanical barrier results suggests a potential relationship between hypoxic stress and damage to the ileal mechanical barrier.

### 3.8. Impact on Gut Microbiota Composition

**Figure 8:**
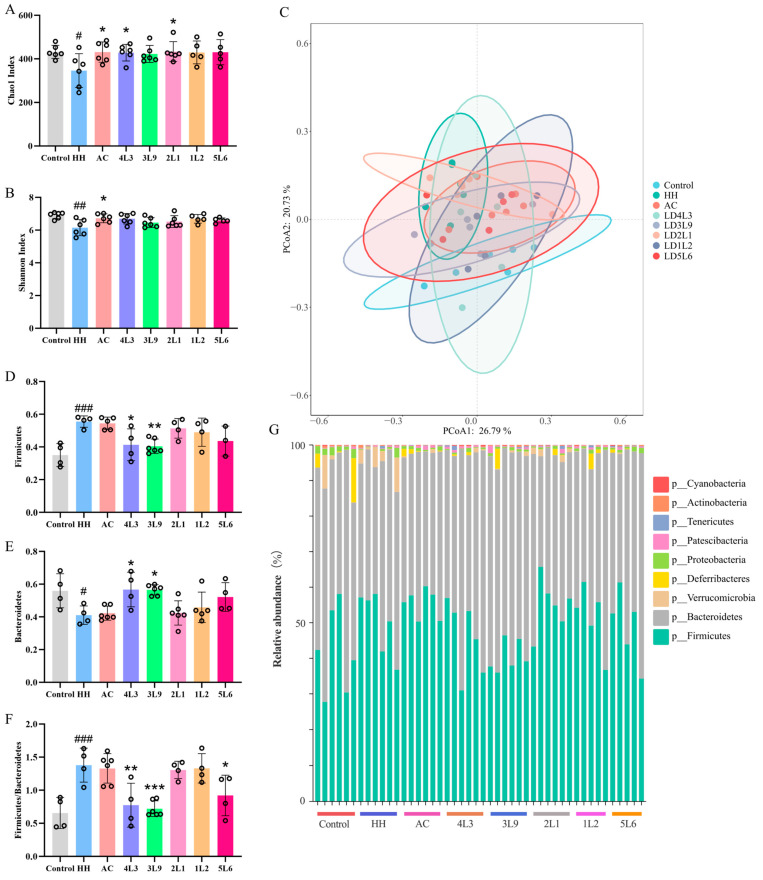
Impact of *Lactobacillus delbrueckii* subsp. *bulgaricus* on gut microbiota diversity and phylum level effects. (**A**) Chao1 index; (**B**) Shannon index; (**C**) PcoA score plot; (**D**) relative abundance of Firmicutes; (**E**) relative abundance of Bacteroidetes; (**F**) Firmicutes/Bacteroidetes ratio; (**G**) relative abundances of gut microbiota at the phylum level (*n* = 4–6). Data are presented as mean ± SD and were analyzed using ANOVA and Dunnett’s test. ^#^ indicates differences between the HH group and the control group, ^#^ *p <* 0.05; ^##^ *p <* 0.01; ^###^ *p <* 0.001. * indicates differences between the intervention group and the HH group, * *p <* 0.05; ** *p <* 0.01; *** *p <* 0.001.

To investigate whether *Lactobacillus delbrueckii* subsp. *bulgaricus* affects the diversity of gut microbiota under hypoxic conditions, the α and β diversity of the mouse gut microbiota were measured. The results showed that the HH group had significantly decreased Chao1 and Shannon indices (*p <* 0.05) (Figure 8A,B), and the AC intervention significantly increased both indices (*p <* 0.05). The 4L3 and 2L1 groups also significantly increased the Chao1 index (*p <* 0.05). Further analysis of the gut microbiota β diversity using PCoA showed significant differences between the HH group and the control group (*p <* 0.001) (Figure 8C), with some recovery in the gut microbiota composition after intervention. Overall, acute hypoxic treatment significantly affected the α and β diversity of the mouse gut microbiota, while intervention with *Lactobacillus delbrueckii* subsp. *bulgaricus* could reverse the changes caused by hypoxia to the gut microbiota.

Further analysis of changes at the phylum level of the mouse gut microbiota showed that it was mainly composed of Firmicutes and Bacteroidetes (Figure 8G), with a significant increase in the Firmicutes/Bacteroidetes ratio in the HH group (*p <* 0.001) (Figure 8F). Interventions with 4L3, 3L9, and 5L6 significantly decreased the F/B ratio (*p <* 0.05) (Figure 8F).

**Figure 9:**
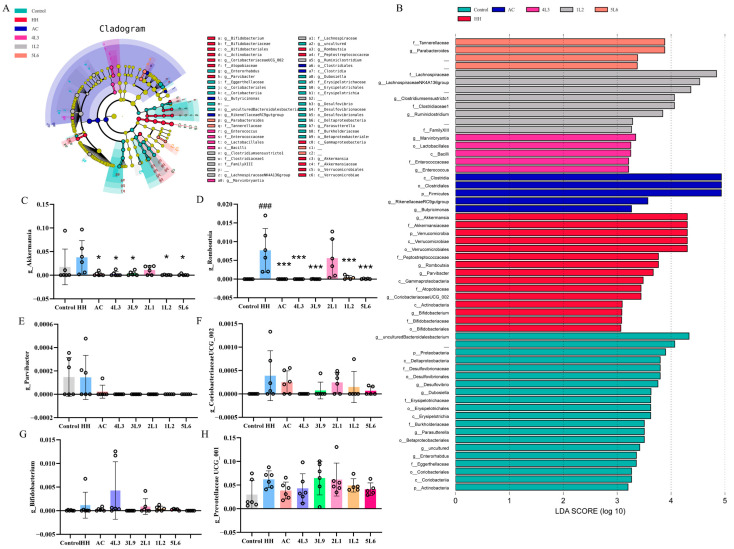
Analysis of characteristic bacterial genera in mouse intestines. (**A**) LefSe analysis; (**B**) distribution histogram based on LDA score (LDA score > 3.0); (**C**) *g_Akkermansia*; (**D**) *g_Romboutsia;* (**E**) *g_Parvibacter*; (**F**) *g_CoriobacteriaceaeUCG_002;* (**G**) *g_Bifidobacterium;* (**H**) *g_Prevotellaceae UCG-001* (*n* = 5–6). Data are presented as mean ± SD and were analyzed using ANOVA and Dunnett’s test. ^#^ indicates differences between the HH group and the control group, ^###^ *p <* 0.001. * indicates differences between the intervention group and the HH group, * *p <* 0.05; *** *p <* 0.001.

To explore the impact of the *Lactobacillus delbrueckii* subsp. *bulgaricus* intervention on the characteristics of gut microbiota, this study also investigated characteristic bacterial genera in different intervention groups based on Linear Discriminant Analysis Effect Size (LEfSe), using an LDA score > 3.0 as a threshold. The results showed that the genera with the most significant changes in the HH group were *Akkermansia*, *Romboutsia*, *Parvibacter*, *CoriobacteriaceaeUCG_002*, and *Bifidobacterium* (Figure 9A,B). Further analysis of the relative abundance of these characteristic genera in different groups showed that the relative abundance of *Romboutsia* significantly increased in the HH group (*p <* 0.001) (Figure 9D), and after intervention, all groups except for the 2L1 group showed a significant decrease in *Romboutsia* relative abundance (*p <* 0.001). A similar pattern was observed for *Akkermansia* relative abundance (Figure 9C), while differences in the relative abundance of *Parvibacter*, *CoriobacteriaceaeUCG_002*, and *Bifidobacterium* among different groups were observed but were not statistically significant.

**Figure 10:**
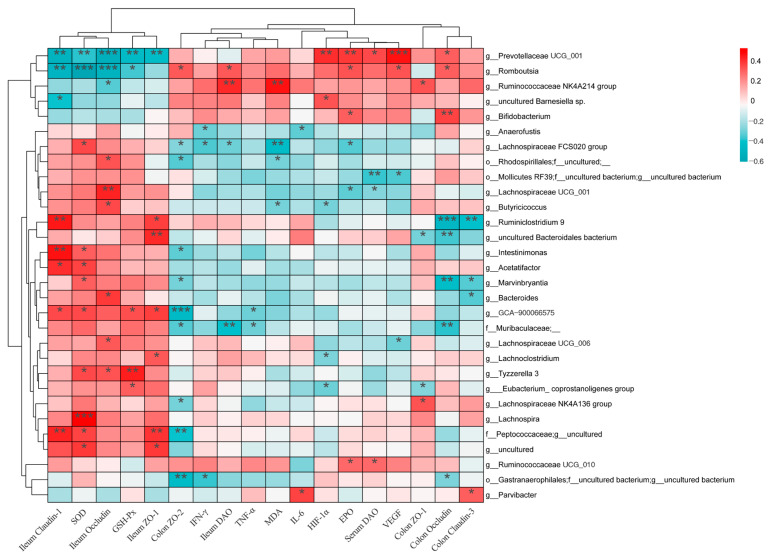
Correlation analysis between gut microbiota and biochemical markers in mice. Spearman correlation coefficients were used for analysis, where * *p <* 0.05; ** *p <* 0.01; *** *p <* 0.001.

Further correlation analysis between the gut microbiota and biochemical markers in mice revealed a strong correlation between *Prevotellaceae UCG-001* and *Romboutsia* with multiple biochemical markers (Figure 10). There was a significant negative correlation with ileal tight junctions and antioxidant enzyme content (*p <* 0.01), and a significant positive correlation with serum hypoxia factors (*p <* 0.05) (Figure 10). Further analysis of the relative abundance of *Prevotellaceae UCG-001* in each group found an increase in the HH group (Figure 9H), while there was a decrease in the AC and 4L3 groups, although not significant. This suggests that *Prevotellaceae UCG-001* and *Romboutsia* play important roles in the process of acute high-altitude response damage, and that the intervention of *Lactobacillus delbrueckii* subsp. *bulgaricus* strain 4L3 may alleviate acute damage by regulating the relative abundance of *Prevotellaceae UCG-001*.

### 3.9. Impact on Fecal Metabolites

The gut is the site of frequent metabolic interactions between microbes and the host, and changes in fecal metabolites can reflect changes in gut microbial metabolism to some extent. Untargeted metabolomics was used to detect metabolite levels in mouse serum. Combining positive and negative ions, data were filtered with mzCloud Best Match ≥ 70, resulting in a total of 177 annotated compounds. Using *p <* 0.05 and |log_2_Fold change| > 1 as criteria, further screening identified 16 significantly upregulated differential metabolites and 23 significantly downregulated differential metabolites in HH compared to the control (Figure S1A); different strains had varying effects on mouse fecal metabolites (Figure S1B–G).

The enrichment analysis of differential metabolites in feces was conducted using the KEGG database, which identified seven closely related pathways: biosynthesis of unsaturated fatty acids, arginine biosynthesis, biosynthesis of phenylalanine, tyrosine and tryptophan, linoleic acid metabolism, fatty acid degradation, phenylalanine metabolism, taurine and hypotaurine metabolism (Figure S2). The biosynthesis pathway of unsaturated fatty acids was enriched with five different unsaturated fatty acids, while the arginine biosynthesis pathway was enriched with two metabolites.

**Figure 11:**
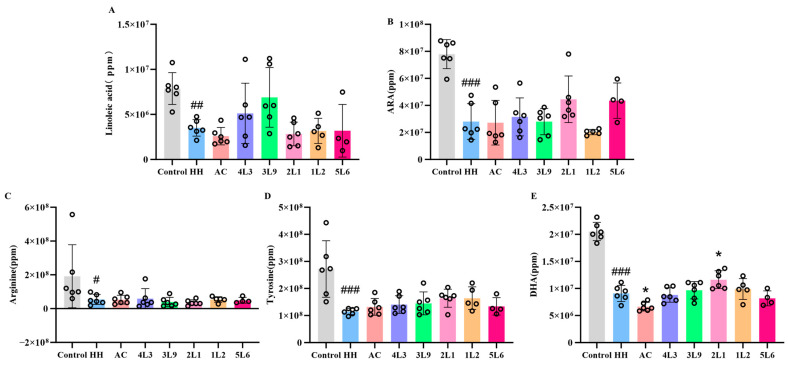
The effect of *Lactobacillus delbrueckii* subsp. *bulgaricus* on mouse fecal metabolites. (**A**) Linoleic acid; (**B**) ARA; (**C**) arginine; (**D**) tyrosine; (**E**) DHA (*n* = 4–6). Data are presented as mean ± SD and analyzed using ANOVA and Dunnett’s test. ^#^ indicates differences between the HH group and the control group, ^#^ *p <* 0.05; ^##^ *p <* 0.01; ^###^ *p <* 0.001. * indicates differences between the intervention groups and the HH group, * *p <* 0.05.

To further understand the specific changes in related substances, the unsaturated fatty acids and amino acid-related substances obtained from pathway enrichment were further analyzed. Hypoxic treatment significantly reduced the content of three types of unsaturated fatty acids in mouse feces (*p <* 0.01) (Figure 11A,B,E), indicating that acute hypoxic exposure affected fatty acid metabolism in mice. Acetazolamide intervention had no significant effect on the content of these unsaturated fatty acids, whereas intervention with *Lactobacillus delbrueckii* subsp. *bulgaricus* resulted in an upward trend in linoleic acid content, although this change was not significant. After hypoxic exposure, the content of tyrosine in mouse feces significantly decreased (*p <* 0.001) (Figure 11D), and the content of arginine decreased but not significantly (Figure 11C). After intervention, both AC and *Lactobacillus* intervention groups showed an upward trend in tyrosine content in feces, but there was no significant change in arginine content.

## 4. Discussion

In acute hypoxic environments, due to the large surface area of the gastrointestinal mucosal organs, they are more susceptible to environmental influences, leading to hypoxia- and ischemia-related diseases [21]. A plausible explanation is that hypoxic conditions can cause damage to the intestinal barrier [22]. Damage to the intestinal barrier can lead to an increase in intestinal permeability. Indeed, it has been discovered that high-altitude environments increase the permeability of the small intestine in healthy individuals [2,23]. When the intestinal barrier is compromised, harmful substances produced by the gut microbiota can enter the bloodstream, triggering local or even systemic inflammatory responses [24]. Hypoxia also has a profound and lasting impact on the gut microbiota [8], and dysbiosis of the gut microbiota can further exacerbate damage to the intestinal barrier [25]. Numerous studies have demonstrated that probiotics or prebiotics can improve intestinal barrier dysfunction caused by hypoxic exposure and reduce inflammatory responses [19,26]. Therefore, this study sought to explore whether lactic acid bacteria, commonly used in everyday life, have the potential to mitigate acute damage induced by hypoxia. Our results confirm that certain strains of *Lactobacillus delbrueckii* subsp. *bulgaricus* have the function of alleviating acute damage caused by hypoxia, including relieving tissue edema, maintaining mechanical barrier function in the ileum, reducing intestinal permeability, decreasing inflammation in mice, and alleviating hypoxic stress. However, there are differences in the alleviating effects of different strains, which may be related to variations in their impact on the intestinal environment and their effectiveness in repairing the intestinal barrier.

Inflammatory responses are a form of self-protection by the body against external stimuli, but excessive inflammation can cause harm to the organism. Various probiotics, such as *Bifidobacterium* and *Lactobacillus rhamnosus*, can alleviate individual inflammatory responses by inhibiting the expression of pro-inflammatory cytokines like IL-12, IFN-γ, TNF-α, and IL-6 [27,28,29]. Inflammatory responses can exacerbate tissue edema by disrupting the integrity of the blood–brain barrier and increasing vascular leakage [30,31]. Our study found that *Lactobacillus delbrueckii* subsp. *bulgaricus* strain 4L3 significantly downregulated the elevation of IL-6 and TNF-α levels induced by hypoxia in mice, thereby reducing inflammation. Extensive research has indicated that there is a mutual promotion between intestinal barrier damage and intestinal inflammation [32], and both IL-6 and TNF-α are also related to the regulation of barrier function [33]. Our results also show that mice with lower levels of inflammation had lower concentrations of DAO in their serum and ileum. DAO, an intracellular enzyme with high activity in the upper villi of the small intestine mucosa, is an important indicator reflecting the integrity of the small intestine’s mechanical barrier [34]. The function of the intestinal barrier depends on the maintenance of tight junction proteins, which are the most important structures forming the mucosal mechanical barrier and include various proteins such as ZO-1, occludin, and claudin. Dou et al. [17] found that bio-selenium nanoparticles synthesized by *Lactobacillus casei* could upregulate ZO-1 and Occludin content reduced by hypoxia, decrease DAO content in the ileum, and protect the intestinal barrier function. Treatment with LGG combined with sucrose also increased the expression of ZO-1 and occludin in the ileum [26]. This suggests that *Lactobacillus* intervention can protect the intestinal barrier by upregulating tight junction proteins in the ileum, reducing harmful substance leakage from the small intestine, and playing a role in lowering inflammation levels. It is worth mentioning that acute hypoxia treatment upregulated the mRNA expression levels of tight junction protein genes in the colon, which differs from the results in the ileum. The reason for this difference is mainly due to the different responses of different intestinal parts to acute hypoxic exposure. It has been reported that the high-altitude environment causes more damage to the small intestine than to the colon [35]. Considering that the transcription levels of the four TJ protein genes in the HH group were increased, this suggests the self-repairing effect of colon cells on tight junction injury caused by acute hypoxia. Under the condition that the cells still have enough self-repair ability, HH group colon cells increased the transcription levels of tight junction proteins to make up for the deficiency of these protein levels. Compared to some effective LAB, the treatment with AC did not adjust the transcription levels of all TJ proteins to levels close to the control group (only the transcription level of ZO-2 was significantly reduced), indicating that the AC group may not have effectively protected the tight junctions (except for ZO-2) in the colon. Conversely, compared to the HH group, some strain intervention groups showed downregulation of TJ protein transcription levels, which precisely suggests that these strains have a protective effect on the tight junction of colon cells. Due to the less severe damage of the tight junction compared to that of HH group, the self-repairing response of colon cells in these groups is not as strong as that in the HH group.

Excessive increases in reactive oxygen species (ROS) can cause damage to individual health. Hypoxic environments disrupt the body’s redox balance, deplete antioxidant enzymes, and further exacerbate oxidative stress damage to the intestinal barrier, compromising its function [36]. Superoxide dismutase (SOD) is an antioxidant enzyme that plays a crucial role in the body’s redox balance. Studies have shown that high-altitude hypoxic environments can lead to a decrease in SOD levels in the body [36]. Malondialdehyde (MDA), as the end product of lipid peroxidation, is one of the common indicators used to measure the degree of oxidative stress [37], while glutathione peroxidase, along with SOD and MDA, is considered an indicator of ROS production. Our study results indicate that AC and 4L3 treatment can significantly alleviate oxidative stress levels in mice, thereby reducing oxidative stress-induced damage to the intestinal barrier [38,39]. Erythropoietin (EPO) stimulates bone marrow to produce red blood cells, increases hemoglobin content, and enhances blood oxygen-carrying and supply capacity. In hypoxic environments, the body releases EPO to cope with the stress caused by hypoxia [40]. Vascular endothelial growth factor (VEGF) is an effective angiogenic and permeability factor, and studies have demonstrated that low-pressure hypoxic exposure can lead to an increase in VEGF levels in individual serum [41]. Hypoxia-inducible factor-1α (HIF-1α) is the active subunit of hypoxia-inducible factor-1 (HIF-1) and is regulated by hypoxia; under hypoxic conditions, HIF-1α levels in the body significantly increase [42]. The levels of these three hypoxia factors in serum can measure the magnitude of hypoxic stress experienced by an individual. *Lactobacillus delbrueckii* subsp. *bulgaricus* can reduce the hypoxic stress on mice by downregulating the levels of hypoxic factors in serum. This result is highly consistent with the findings regarding the ileal mechanical barrier in mice, suggesting that hypoxic stress in mice may be related to damage to the ileal mechanical barrier. It is worth noting that the 2L1 strain showed inconsistent deterioration or protective effects on some appearance indexes and biochemical indexes after treating model mice. This contradiction is mainly due to the fact that LAB are complex organisms. This is different from the impact of some compounds with clear molecular structure on the body. Obviously, the impact of LAB on the body will be more complex, which is manifested in the fact that the impact on the same index may come from multiple pathways or mechanisms of LAB or the impact of the same strain on the host may be multifaceted. That is, although a LAB strain shows a positive protective effect on some biochemical indicators, its comprehensive effect on the host (apparent indicators) may be invalid or even harmful due to the existence of other effects from this complex organism.

The gut microbiome plays a significant role in human life, participating in various vital activities such as nutrient metabolism, immune regulation, and disease prevention [43]. Our findings indicate that 4L3 can modulate the ratio of Firmicutes to Bacteroidetes, thereby ameliorating dysbiosis in hypoxic mice [44]. Further correlation analysis revealed that *Prevotellaceae UCG-001* and *Romboutsia* are significantly negatively correlated with ileal barrier integrity and antioxidant enzyme levels, and positively correlated with serum hypoxia factors. This suggests that *Prevotellaceae UCG-001* and *Romboutsia* play an important role in the damage caused by acute hypoxia in mice and are closely related to the intestinal barrier. Multiple studies have found an increase in the abundance of *Prevotellaceae UCG-001* in diseases associated with inflammation [45,46,47], and reducing its relative abundance can effectively alleviate inflammatory symptoms. This indicates that *Prevotellaceae UCG-001* may also be involved in the inflammation caused by hypoxia-induced acute injury, and modulating the relative abundance of *Prevotellaceae UCG-001* can indeed alleviate the inflammatory response in acute hypoxic mice.

Fecal metabolites can reflect changes in microbial metabolites in the gut to a certain extent and can more intuitively demonstrate the role of the gut microbiota in the host metabolism. A previous study found that unsaturated fatty acids are high-affinity ligands for hypoxia-inducible factor 3α, mediating cellular responses to hypoxia [48]. Oka et al. [49] found that the concentration of linoleic acid in the hearts of mice with chronic hypoxia was lower, a change that may be related to alterations in the body’s oxidative stress levels. This suggests that unsaturated fatty acids may play an antioxidative role in high-altitude response damage induced by hypoxia. Functional amino acids, such as arginine, glutamate, or glutamine, can help enhance intestinal mucosal immunity, reduce oxidative damage, and strengthen intestinal barrier function [50]. Our results confirm that hypoxic stimulation reduces the levels of arginine and tyrosine in mouse feces. Although we currently lack clear evidence of arginine’s positive role in hypoxia-induced acute injury, the overall trend allows us to see differences in arginine and tyrosine levels in the intervention group treated with *Lactobacillus delbrueckii* subsp. *bulgaricus*. The existing research also confirms that injecting arginine can reduce patients’ AMS scores [51] and alleviate mean pulmonary artery pressure in rat lung tissue caused by acute hypobaric hypoxia [52]. It is necessary to precisely detect the levels of arginine and tyrosine in mouse feces through targeted metabolomic approaches next, to explore their relationship with acute hypoxic injury in mice.

Our research found that intervention with *Lactobacillus delbrueckii* subsp. *bulgaricus* effectively alleviated various injuries caused by acute hypoxic exposure, including inflammatory response, intestinal barrier damage, gut microbiota dysbiosis, and metabolite changes. This gives us a positive signal that the alleviation of plateau-related diseases by lactobacilli is practicable, which lays an important theoretical foundation for the future development of probiotics targeting plateau-related diseases, and also provides an important theoretical basis for the development of dietary strategies to reduce plateau-related diseases in the future.

## 5. Conclusions

In summary, acute hypoxic exposure induces severe oxidative stress responses, inflammation, intestinal barrier damage, dysbiosis of the gut microbiota, and changes in fecal metabolites in mice. Intervention with certain strains of *Lactobacillus delbrueckii* subsp. *bulgaricus* can enhance the integrity of tight junctions in the intestinal epithelium to varying degrees, alleviate oxidative stress, reduce inflammatory responses, and reshape the gut homeostasis to mitigate the hypoxic stress experienced by mice and lessen the damage caused by acute hypoxia. There are differences in the alleviation effects between different strains, which, from a correlation perspective, may be related to the strains’ ability to regulate the abundance of *Prevotellaceae UCG-001* in the gut. This study has revealed that *Lactobacillus delbrueckii* subsp. *bulgaricus* strain 4L3 may alleviate acute hypoxic damage in mice by protecting intestinal barrier functions and suppressing the abundance of harmful bacteria in the gut, suggesting that probiotics have great potential as a future target for mitigating damage caused by acute high-altitude sickness.

## Figures and Tables

**Table 1:** Information of experimental strains.

Number in Article	Strain Number	Species	Strain Source
1L2	DQHXNS1L21	*Lactobacillus. Delbrueckii* subsp. *bulgaricus*	Dairy products
2L1	DQHXNS2L1	*Lactobacillus. Delbrueckii* subsp. *bulgaricus*	Dairy products
3L9	DQHXNS3L9	*Lactobacillus. Delbrueckii* subsp. *bulgaricus*	Dairy products
4L3	DQHXNS4L3 (CCFM1387)	*Lactobacillus. Delbrueckii* subsp. *bulgaricus*	Dairy products
5L6	DQHXNS5L6	*Lactobacillus. Delbrueckii* subsp. *bulgaricus*	Dairy products

**Table 2:** The primers sequence.

Primer Name	Sequence (5′-3′)
*β-actin*	F: CCTTCCAGCAGATGTGGATCA R: CTCAGTAACAGTCCGCCTAGAA
Occludin (*OCLN*)	F: CCCCAATGTTGAAGAGTGGGTTA R: CACACTCAAGGTCAGAGGAATCT
ZO-1 (*TJP1*)	F: CTCAAGTTCCTGAAGCCCGT R: GCAAAAGACCAACCGTCAGG
ZO-2 (*TJP2*)	F: ATGGGAGCAGTACACCGTGA R: GCTGAACGGCAAACGAATGG
Claudin-3 (*CLDN3*)	F: ACCAACTGCGTACAAGACGAG R: CGGGCACCAACGGGTTATAG

## Data Availability

The datasets generated and analyzed during the current study are available from the corresponding author. The data are not publicly available due to privacy reasons.

## Supplementary Materials

The following supporting information can be downloaded at: https://www.mdpi.com/article/10.3390/nu16101465/s1, Figure S1: the effect of *Lactobacillus delbrueckii* subsp. *bulgaricus* on mouse fecal metabolites. (A–G) Differential metabolites between control, AC, 4L3, 3L9, 2L1, 1L2, 5L6, and HH groups; Figure S2: KEGG pathway enrichment analysis of differential metabolites.
