# Supplementary material for: Lactobacillus delbrueckii subsp. bulgaricus Alleviates Acute Injury in Hypoxic Mice
Source: Nutrients. 2024 May 13;16(10):1465. doi: 10.3390/nu16101465 (PMC11124140; doi:10.3390/nu16101465)
Supplement: Supplementary file 1 [file nutrients-16-01465-s001.zip › nutrients-2987312-supplementary.pdf]

Supplementary materials

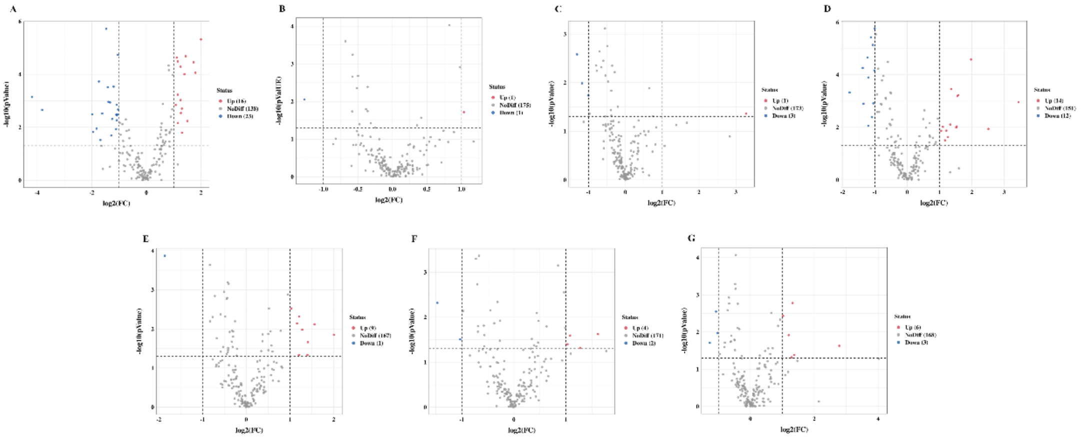

Figure S1 The effect of *Lactobacillus delbrueckii subsp. bulgaricus* on mouse fecal metabolites. (A-G) Differential metabolites between Control, AC, 4L3, 3L9, 2L1, 1L2, 5L6, and HH groups,

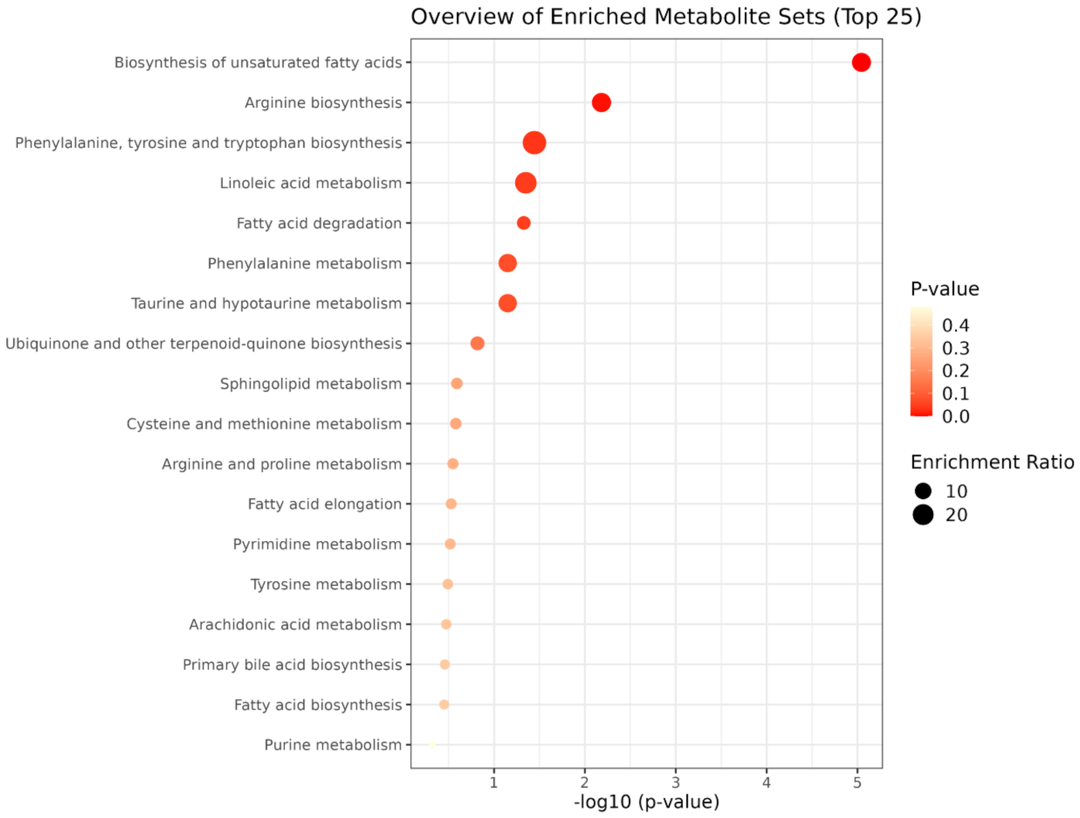

Figure S2 KEGG pathway enrichment analysis of differential metabolites.
